# Thyrotropin-producing pituitary adenoma simultaneously existing with Graves’ disease: a case report

**DOI:** 10.1186/s13256-016-1172-4

**Published:** 2017-01-06

**Authors:** Nobuhiko Arai, Makoto Inaba, Takamasa Ichijyo, Hiroshi Kagami, Yutaka Mine

**Affiliations:** 1 Department of Neurosurgery, Saiseikai Yokohamashi, Tobu Hospital, Yokohama, Japan; 2 Department of Endocrinology and Metabolism, Saiseikai Yokohamashi, Tobu Hospital, Yokohama, Japan

**Keywords:** Graves’ disease, Pituitary neoplasms, Thyrotropin, Endoscopy, Neurosurgery, TSHoma, TSH-producing tumor

## Abstract

**Background:**

Thyrotropin-producing pituitary tumor is relatively rare. In particular, concurrent cases associated with Graves’ disease are extremely rare and only nine cases have been reported so far. We describe a case of a thyrotropin-producing pituitary adenoma concomitant with Graves’ disease, which was successfully treated.

**Case presentation:**

A 40-year-old Japanese woman presented with mild signs of hyperthyroidism. She had positive anti-thyroid-stimulating hormone receptor antibody, anti-thyroglobulin antibody, and anti-thyroid peroxidase antibody. Her levels of serum thyroid-stimulating hormone, which ranged from low to normal in the presence of high levels of serum free thyroid hormones, were considered to be close to a state of syndrome of inappropriate secretion of thyroid-stimulating hormone. Magnetic resonance imaging showed a macropituitary tumor. The coexistence of thyrotropin-producing pituitary adenoma and Graves’ disease was suspected. Initial therapy included anti-thyroid medication, which was immediately discontinued due to worsening symptoms. Subsequently, surgical therapy for the pituitary tumor was conducted, and her levels of free thyroid hormones, including the thyroid-stimulating hormone, became normal. On postoperative examination, her anti-thyroid-stimulating hormone receptor antibody levels decreased, and the anti-thyroglobulin antibody became negative. The coexistence of thyrotropin-producing pituitary adenoma and Graves’ disease is rarely reported. The diagnosis of this condition is complicated, and the appropriate treatment strategy has not been clearly established.

**Conclusions:**

This case suggests that physicians should consider the coexistence of thyrotropin-producing pituitary adenoma with Graves’ disease in cases in which thyroid-stimulating hormone values range from low to normal in the presence of thyrotoxicosis, and the surgical treatment of thyrotropin-producing pituitary adenoma could be the first-line therapy in patients with both thyrotropin-producing pituitary adenoma and Graves’ disease.

## Background

Thyrotropin-producing pituitary adenoma (TSHoma) is a very rare disease, representing less than 1% of all pituitary tumors [1, 2]. Graves’ disease is a common autoimmune endocrinological disorder. Although these diseases manifest similar symptoms of thyrotoxicosis, the treatment for each disease is very different. Up to now (August 2016), nine cases of TSHoma associated with Graves’ disease have been reported [3–10]. In some of those reports, the coexistence of these two diseases made the diagnosis and treatment complicated; thus, thyrotoxicosis was insufficiently controlled. Here we describe an extremely rare case of TSHoma simultaneously existing with Graves’ disease in which an anti-thyroid drug exacerbated the patient’s symptom; however, pituitary surgery was successful.

## Case presentation

A 40-year-old Japanese woman presented to a hospital because of a headache in 2004. A magnetic resonance image (MRI) showed one adenoma on her pituitary gland that measured 4×7 mm. Her levels of pituitary anterior lobe hormones were normal. Thus, the tumor was thought to be non-functioning. Thereafter, the tumor was followed yearly with MRI to determine whether it was growing. In September 2009, periodic MRI showed that the pituitary tumor had become larger. She was referred to our hospital for further evaluation. She had no remarkable history or medications. She had a family history of rheumatoid arthritis. On physical examination, her blood pressure was 127/75 mmHg, and her pulse was regular at 105 beats/minute. Her body temperature was 36.8 °C, height was 163.7 cm, body weight was 53.8 kg, and body mass index was 20.1 kg/m^2^. Her consciousness was alert. She had no neck goiter or leg edema. Both Achilles’ tendon reflexes were moderately increased. She had mild exophthalmos. She denied any headaches or visual field defect. Her other clinical symptoms were not notable. An endocrinological examination and thyroid echocardiography were performed because of hyperthyroidism; in particular, Graves’ disease was suspected. Her serum free triiodothyronine (FT3) level was 6.3 pg/mL (normal 2.3 to 4.0 pg/mL), free thyroxine (FT4) level was 2.3 ng/dL (normal 0.9 to 1.7 ng/dL), thyroid-stimulating hormone (TSH) level was 0.27 μIU/mL (normal 0.5 to 5.0 μIU/mL), anti-TSH receptor antibody (TRAb) was positive (5.0 IU/L, normal <1.0 IU/L), anti-thyroglobulin antibody (TgAb) was positive (2.3 IU/mL, normal <0.3 IU/mL), and anti-thyroid peroxidase antibody (TPOAb) was positive (4.6 IU/mL, normal <0.3 IU/mL). Ultrasonography showed a moderately hypervascular thyroid without any nodes. Due to the increase in her FT3 and FT4 levels, and the decrease in her TSH level and TRAb positivity, Graves’ disease was diagnosed, and anti-thyroid drug therapy was started in April 2010. Subsequently, she stopped taking her medication because she felt that the headache and sweating were exacerbated. In Graves’ disease, the TSH level is normally suppressed by negative feedback due to an increase in the FT3 and FT4 levels; the TSH value falls below the detectable level (<0.1 μIU/mL according to second-generation TSH chemiluminometric assays). In our case, our patient’s TSH value ranged from 0.1 to 0.5 for the 6 months before she started the medication. We suspected that this case was close to being in a state of syndrome of inappropriate secretion of TSH (SITSH), although in SITSH, the TSH values are generally increased.

**Fig. 1:**
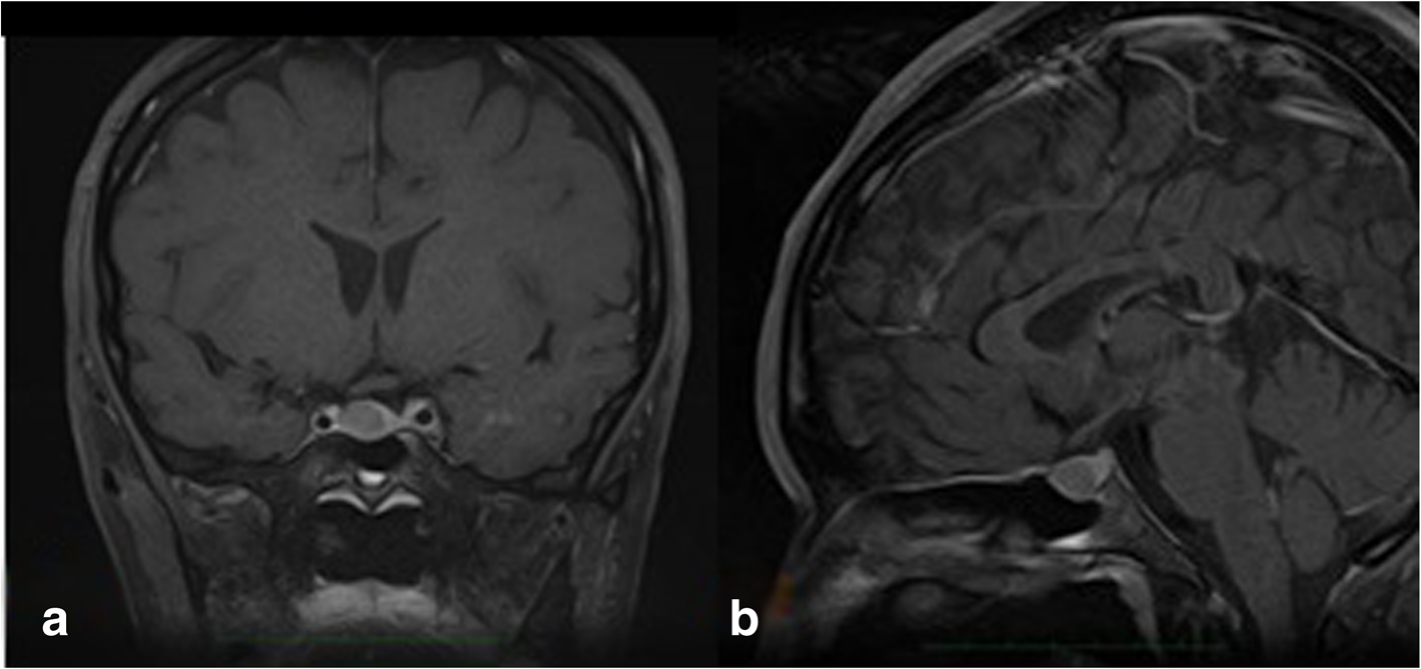
Imaging study. **a** T1-gadolinium magnetic resonance imaging on admission showing a less enhanced area in the sella turcica, which seemed to be a macroadenoma (10×13 mm). **b** The tumor slightly invaded the right lateral cavernous sinus

The simultaneous coexistence of TSHoma was highly suspected. A pituitary MRI showed a less enhanced area in the sella turcica (10×13 mm). On graphical examination, the tumor appeared to be slightly invading her right lateral cavernous sinus (Fig. 1). An anterior pituitary hormone stimulation test indicated that only the TSH had an impaired response to a TSH-releasing hormone (TRH) stimulation test.

**Fig. 2:**
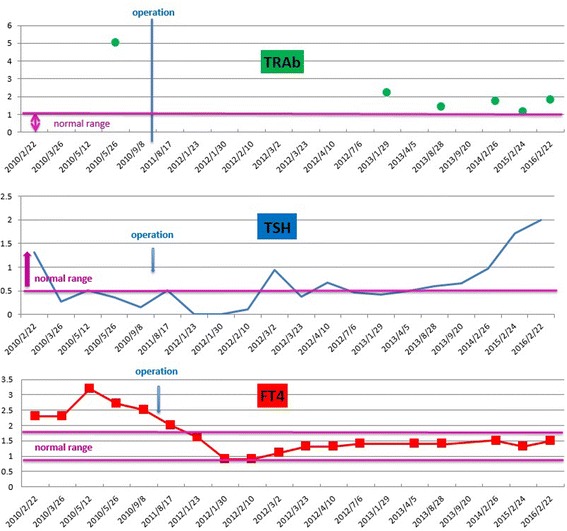
Levels of the anti-thyroid-stimulating hormone receptor antibody, thyroid-stimulating hormone, and free thyroxine. The free thyroxine level was above 1.5 ng/dL preoperatively. Total resection of the pituitary adenoma by endoscopic trans-sphenoidal neurosurgery was performed in January 2011. The free thyroxine level decreased within normal range. Subsequently, the euthyroid status was maintained. In the meantime, the thyroid-stimulating hormone values ranged from 0.1 to 0.5 μIU/mL preoperatively. The thyroid-stimulating hormone value of a typical patient with Basedow’s disease is less than 0.1 μIU/mL. In the case of syndrome of inappropriate secretion of thyroid-stimulating hormone, the thyroid-stimulating hormone value is more than 0.5 μIU/mL. The present case did not fit either of these two diseases. On postoperative examination, the thyroid-stimulating hormone value decreased below the detection threshold. Gradually, the thyroid-stimulating hormone values became normal. Anti-thyroid-stimulating hormone receptor antibody was continuously above the upper limit of normal range. *FT4* free thyroxine, *TRAb* anti-thyroid-stimulating hormone receptor antibody, *TSH* thyroid-stimulating hormone

**Fig. 3:**
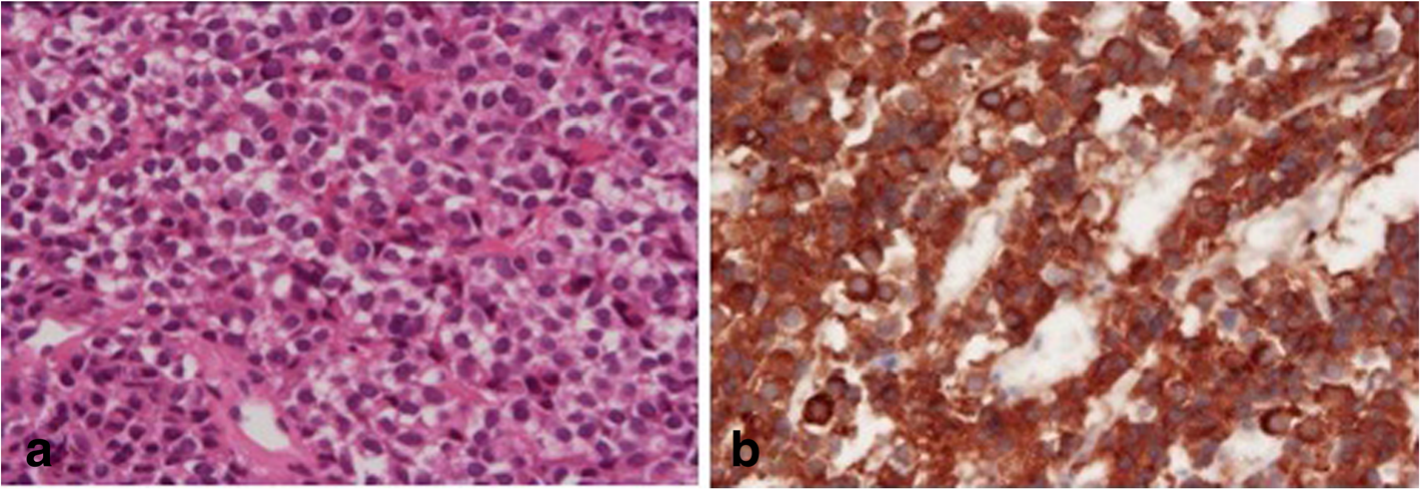
Pathological diagnosis of pituitary adenoma was determined (**a**). Immunostainning showed the following: thyroid-stimulating horomone, strongly positive (**b**); follicle stimulating hormone, weakly positive; luteinizing hormone, negative; growth hormone, focally positive: prolactin, focally positive; and adenocotrophic hormone, negative

Endoscopic trans-sphenoidal neurosurgery was performed for the pituitary adenoma in January 2011. Immediately postoperatively, her TSH values decreased below the detection limit (<0.1 μIU/mL) [11], and her FT3 and FT4 levels became within normal range. A histological examination showed that immunostaining was 99% positive for TSH-β (Figs. 2 and 3). Postoperatively, her TSH levels gradually became normal, and her FT3 and FT4 levels did not increase until February 2016. Her symptom disappeared. TRAb continued to be positive; however, her level of TRAb decreased. TgAb became negative, whereas TPOAb continued to be positive.

## Discussion

TSHomas are a rare cause of hyperthyroidism, and they account for less than 1% of all pituitary adenomas [1, 2, 10]. They often manifest with mild symptoms of thyrotoxicosis, and they are associated with abnormal levels of thyroid hormones and TSH [10]. Although the levels of FT3 and FT4 increase, the TSH levels are not suppressed. This state is defined as SITSH [12]. In general, SITSH indicates the possibility of TSHoma and syndrome of resistance to thyroid hormone (RTH). The mechanism of RTH is a thyroid hormone receptor (TR)-β deficit. We analyzed the TR-β mutations of our patient, but they were negative. On postoperative examination, her TSH values decreased below the detection limit, and histological examination showed that immunostaining was 99% positive for TSH-β. The present case could definitely be diagnosed as having TSHoma. However, two points were unusual in our case. First, our patient was TRAb positive. TRAb provokes the TSH receptor and produces more amounts of the thyroid hormone than that of a healthy person. The determination of TRAb and exophthalmos found on the initial examination provided definite evidence for Graves’ disease [10]. Second, preoperatively, the TSH levels in our case ranged from normal to low, although in typical cases of SITSH, they range from normal to high [13, 14]. We consider that this obscure value is the result of concomitant Graves’ disease, and hyperthyroidism in this case was attributed to both TSHoma and Graves’ disease.

TSHoma associated with Graves’ disease is extremely rare. We searched PubMed for cases of TSHoma associated with autoimmune hyperthyroidism, Graves’ disease. Only nine cases (written in English) have been reported so far (August 2016) [3–10]. In four cases, TSHoma preceded the occurrence of Graves’ disease [3, 4, 6, 7]. In two cases, Graves’ disease occurred prior to the onset of TSHoma [8, 9]. In the other three cases, Graves’ disease and TSHoma probably coexisted at the initial diagnosis, as in our case [5, 8, 10].

The treatment for TSHoma concomitant with Graves’ disease is complicated, because the treatment of Graves’ disease is very different from that of TSHoma. As previously mentioned, three cases with both TSHoma and Graves’ disease have been reported. In all these cases, anti-thyroid medication was chosen first to treat Graves’ disease. However, the medication failed to normalize the FT4, FT3, and TSH levels, resulting in the progression of TSHoma. The reason for this is because anti-thyroid therapy inhibits negative feedback to TSH, just as anti-thyroid medication administered under a misdiagnosis of Graves’ disease may carry the risk of promoting TSHoma due to the positive feedback system [15]. Therefore, anti-thyroid therapy should not be considered the first-choice treatment in patients with both TSHoma and Graves’ disease.

In our case, anti-thyroid medication was started first; however, our patient soon discontinued the medication of her own accord. Eventually, TSHoma was successfully treated by endoscopic trans-sphenoidal neurosurgery. Subsequently, her TSH value decreased below the detectable minimum limit, and it gradually normalized. Her FT3 and FT4 levels became normal immediately postoperatively. Her subjective symptom disappeared. Of interest, her TRAb levels decreased, TgAb became negative, and Graves’ hyperthyroidism has not occurred since the operation. The cause of postoperative remission of Graves’ hyperthyroidism is not clear. However, these observations suggest that the treatment for TSHoma might have improved Graves’ hyperthyroidism in our case, although the most probable explanation was considered to be that Graves’ disease became euthyroid state spontaneously.

TSH plays an important role in the maintenance of normal physiology and in the regulation of immunomodulatory gene expression of thyrocytes [7]. One explanation for these observations is that the abnormal hypersecretion of TSH can produce anti-idiotypic antibodies, causing Graves’ disease [6], and the normalization of TSH secretion by treating TSHoma may reduce the production of those antibodies and thus improve Graves’ hyperthyroidism.

In any case, the surgical treatment of TSHoma may be considered the first choice in patients with both TSHoma and Graves’ disease to prevent the risk of promoting TSHoma by anti-thyroid therapy. Conversely, some reports have shown that Graves’ disease has subsequently occurred after TSHoma is treated. In one case, it was suggested that a rapid reduction in the TSH levels postoperatively induced apoptosis and activated an autoimmune response against the thyroid gland. The mechanism is the increased expression of various cell surface markers on thyrocytes [7]. Hence, physicians should pay attention to the development of Graves’ disease after TSHoma is treated, especially in cases with concomitant Graves’ disease.

## Conclusions

The present case demonstrated that trans-sphenoidal surgery, which is the standard therapy for TSHoma, should be considered first-line therapy in patients with both TSHoma and Graves’ disease. Then, when Graves’ disease becomes active, anti-thyroid therapy should be started as second-line therapy. Moreover, a definitive operation is needed to prevent the subsequent treatment from becoming complicated.

## Abbreviations

FT3: Free triiodothyronine
FT4: Free thyroxine
MRI: Magnetic resonance image
RTH: Resistance to thyroid hormone
SITSH: Syndrome of inappropriate secretion of TSH
TgAb: Anti-thyroglobulin antibody
TPOAb: Anti-thyroid peroxidase antibody
TR: Thyroid hormone receptor
TRAb: Anti-thyroid-stimulating hormone receptor antibody
TRH: Thyroid-stimulating hormone-releasing hormone
TSH: Thyroid-stimulating hormone
TSHoma: Thyrotropin-producing pituitary adenoma
